# Weniger Operationen und Kosten wegen Rückenschmerzen in einem Versorgungsprogramm mit interdisziplinärem Zweitmeinungsverfahren: Eine kontrollierte, nichtrandomisierte Interventionsstudie mit GKV-Daten

**DOI:** 10.1007/s00482-022-00624-2

**Published:** 2022-02-22

**Authors:** Christoph J. Wagner, Gabriele Lindena, Grit Ayyad, Andrea Otzdorff, Ulf Marnitz, Karen Bienek, Björn von Pickardt, Stephanie Sehlen, Werner Wyrwich

**Affiliations:** 1 AOK Nordost, Wilhelmstraße 1, 10963 Berlin, Deutschland; 2 CLARA Clinical Analysis Research and Application, Clara-Zetkin-Straße 34, 14532 Kleinmachnow, Deutschland; 3 Rückenzentrum am Markgrafenpark GmbH, Markgrafenstraße 19, 10969 Berlin, Deutschland; 4 Reha-Tagesklinik im Forum Pankow GmbH und Co. KG, Hadlichstraße 19, 13187 Berlin, Deutschland; 5 Reha-Zentrum Teltow GmbH und Co. KG, Oderstraße 69, 14513 Teltow, Deutschland

**Keywords:** Planbare Operation, Wirbelsäulenoperation, Chronischer Rückenschmerz, Schmerztherapie, Krankheitskosten, Elective surgery, Spine surgery, Chronic back pain, Pain therapy, Cost of illness

## Abstract

**Hintergrund:**

Für interdisziplinäre Zweitmeinungsverfahren (IZMV) bei empfohlenen Rückenoperationen (ROP) fehlen Wirksamkeitsbelege. Die AOK Nordost bietet seit 2015 das Versorgungsprogramm RückenSPEZIAL an. Es enthält Vorbefundprüfung, IZMV und optional eine interdisziplinär-multimodale Schmerztherapie (IMST). Ziel dieser Studie ist die Ermittlung der Wirksamkeit von RückenSPEZIAL für ROP und rückenschmerzbezogene Kosten (RBK) im Vergleich zu Patienten mit ROP-Empfehlung ohne RückenSPEZIAL.

**Methoden:**

Versicherte der AOK Nordost legten im AOK-Servicecenter einen ROP-Krankenhauseinweisungsschein vor und erhielten eine dokumentierte Beratung zur RückenSPEZIAL-Teilnahme. Nach einer 1:1-Matched-pairs-Ziehung wurden Patienten, die nach dieser Beratung (= Stichtag) an RückenSPEZIAL teilnahmen (Interventionsgruppe [IG]), mit Patienten verglichen, die nach der Beratung nicht teilnahmen (Vergleichsgruppe [VG]). Patientenmerkmale, ROP und RBK wurden aus Daten der AOK Nordost operationalisiert.

**Ergebnisse:**

Von 108 IG-Patienten und 108 VG-Patienten hatten 34 (42 %) weniger IG-Patienten eine oder mehr ROP in 365 Folgetagen (relatives Risiko [RR] 0,58; *p* < 0,001). Die Subgruppenanalyse zeigte für 21 IG-Patienten mit IZMV und IMST ein RR von 0,13 (*p* < 0,001) und für 67 IG-Patienten mit alleinigem IZMV ohne IMST ein RR von 0,59 (*p* < 0,001). Die Veränderung der RBK von Vorjahr auf Folgejahr war für die IG um 50,2 %-Punkte geringer als für die VG (*p* = 0,088).

**Diskussion:**

Die ROP-Unterschiede zugunsten des Selektivvertrags RückenSPEZIAL waren signifikant (*p* < 0,05). Für die spezifische Population ist eine Refinanzierung der Interventionskosten von RückenSPEZIAL tendenziell erwartbar (näherungsweise signifikant, geringe Fallzahl). Es ist eine Verzerrung aufgrund von Selbstselektion anzunehmen.

**Zusatzmaterial online:**

Die Online-Version dieses Beitrags (10.1007/s00482-022-00624-2) enthält weitere Tabellen.

## Abstract (en)

**Background:**

There is no evidence of effectiveness for interdisciplinary second opinion procedures (ISOP) for recommended back surgery (BS). Since 2015, AOK Nordost has been offering the care program RückenSPEZIAL comprising a preliminary examination, ISOP, and optional interdisciplinary multimodal pain therapy (IMPT). The objective of this study is to determine the effectiveness of RückenSPEZIAL to reduce BS and back pain-related costs (BPRC) compared to patients who likewise received a recommendation for back surgery but not RückenSPEZIAL.

**Methods:**

Insured persons in the AOK Nordost consulted the AOK service center, presented a BS hospital admission slip and received advice to participate in RückenSPEZIAL. Following a 1:1 “matched pairs” selection, patients who participated in RückenSPEZIAL (intervention group [IG]) after this consultation (reference date) where compared with patients who did not participate after this consultation (comparison group [CG]). Patient characteristics, BS and BPRC were operationalised from AOK Nordost claims data.

**Results:**

Of 108 IG patients and 108 CG patients, 34 (42%) fewer IG patients had one or more BS in 365 following days (relative risk [RR] 0.58; *p* < 0.001). The subgroup analysis showed for 21 IG patients with ISOP and IMPT an RR of 0.13 (*p* < 0.001), and for 67 IG patients with solely ISOP without IMPT an RR of 0.59 (*p* < 0.001). The increase in RBC from the previous year to the following year was 50.2 percentage points lower for IG patients compared to CG patients (*p* = 0.088).

**Discussion:**

The differences in BS were significant (*p* < 0.05) and in favor of RückenSPEZIAL. For the specific population it can be expected that mainly savings on BS can cover the intervention costs of RückenSPEZIAL (approximately significant, small case number). Bias due to self-selection needs to be assumed.

## Einleitung

Versicherte der gesetzlichen Krankenversicherung (GKV) haben seit dem GKV-Versorgungsstärkungsgesetz 2015 in Deutschland einen Anspruch auf ärztliche Zweitmeinung vor bestimmten planbaren Operationen (§ 27b SGB V). Eingriffe an der Wirbelsäule wurden allerdings erst mit Inkrafttreten zum 19.11.2021 in die Liste der anspruchsberechtigenden Operationen aufgenommen [5]. Seit 2015 nutzen daher Krankenkassen wie die AOK Nordost Verträge zur besonderen Versorgung (§§ 140 SGB V), um Ihren Versicherten selbst konzipierte Zweitmeinungsverfahren bei Rückenoperationen (ROP) anzubieten und diese auch zu evaluieren.

In 2018 gab es in Deutschland 815.295 ROP-Krankenhausfälle mit Operationen- und Prozedurenschlüssel (OPS) Kapitel 5-83 [2]. In 2018 kostete ein solcher ROP-Krankenhausfall bei der AOK Nordost durchschnittlich 8960 €. Es gibt Hinweise, dass ROP teils keinen Nutzen generieren und teils aufgrund wirtschaftlicher Anreize des Krankenhaus-Vergütungssystems durchgeführt werden [8].

Für ROP fehlen bislang kontrollierte Studien zu Zweitmeinungsverfahren, die in einer Nachbeobachtung die tatsächlich erfolgten Rückenoperationen (ROP) sowie die rückenschmerzbezogenen Kosten (RBK) mit einer Vergleichsgruppe analysieren [1, 9, 11]. Die Information zu einer ROP-Erstempfehlung liegt in der Regel nicht systematisch in den Abrechnungsdaten der GKV vor. In dieser Studie gelang es, ROP-Erstempfehlungen systematisch über ärztliche Krankenhauseinweisungsscheine zu identifizieren, die der AOK Nordost vorgelegt wurden. Diese Informationsbasis ermöglichte einen Matched-pairs-Vergleich von Versicherten mit einer ROP-Erstempfehlung „mit“ versus „ohne“ besondere Versorgung mit einem Zweitmeinungsverfahren.

**Figure 1:**
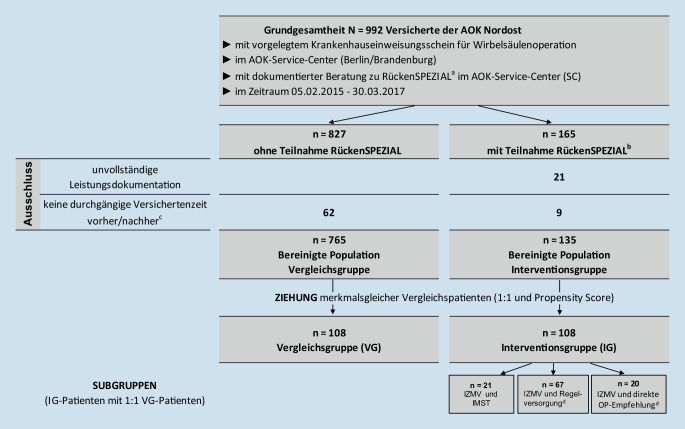

Die AOK Nordost bietet ihren Versicherten seit dem 15.04.2015 das Versorgungsprogramm RückenSPEZIAL als Vertrag über eine besondere Versorgung nach §§ 140a ff. SGB V an. Versicherte mit Krankenhauseinweisungsschein für eine ROP können teilnehmen und erhalten in 5 ambulanten Rückenzentren in Berlin und Brandenburg eine Vorbefundprüfung, ein interdisziplinäres Zweitmeinungsverfahren (IZMV) sowie auf Empfehlung optional eine interdisziplinär-multimodale Schmerztherapie (IMST). In der Vorbefundprüfung durch den Arzt werden fakultative Ausschlusskriterien zur Orientierung geprüft (siehe Tab. 2). Das IZMV ist zentrale Vertragsleistung. Es enthält ein ca. dreistündiges Assessment mit ärztlicher Diagnostik, schmerzpsychologischem Screening und physiotherapeutischer Befunderhebung, eine interdisziplinäre Fallkonferenz mit Einbindung/Kontaktierung des Erstmeiners, ein Versichertengespräch mit individueller Therapieempfehlung sowie die Arztbrieferstellung. Auf Empfehlung des IZMV hin ergeben sich drei Subgruppen der Weiterbehandlung nach dem IZMV (Abb. 1):

1. Der Patient erhält auf ärztliche Empfehlung hin die zusätzliche RückenSPEZIAL-Leistung IMST („IZMV und IMST“).
2. Der Patient wird ohne weitere RückenSPEZIAL-Leistungen mit einem Arztbrief, Empfehlungen und oftmals einem Telefonat mit dem Erstmeiner zurück in die Therapie der ambulanten Regelversorgung entlassen („IZMV und Regelversorgung“).
3. Der Patient erhält eine direkte OP-Empfehlung („IZMV und direkte OP-Empfehlung“).

Im Rahmen der IMST [7] erbringen in einem ambulanten Versorgungssetting Fachärzte für Orthopädie, physikalische und Rehabilitationsmedizin, Anästhesiologie, Ärzte mit Zusatzqualifikation Schmerztherapeut, Psychotherapeuten, Sozialtherapeuten, Physiotherapeuten, Sporttherapeuten, Ergotherapeuten je nach Versichertensituation drei mögliche Leistungspakete: (i) Vollzeitteilnahme für 100 h in 20 Tagen, (ii) Vollzeitteilnahme für 60 h in ≤ 4 Wochen, (iii) berufsbegleitende Teilnahme für 40–50 h in ≤ 3 Monaten. Die IMST umfasst als ambulante modulare Gruppentherapie die Komponenten Schmerztherapie, Edukation, Psycho-, medizinische Trainings- und Ergotherapie sowie regelmäßige interdisziplinäre Fallkonferenzen.

Ziel dieser Studie ist es, die Wirksamkeit des Vertrags RückenSPEZIAL hinsichtlich einer Reduktion der ROP und RBK im Folgejahr zu analysieren. Verglichen wird mit Patienten, die gleichermaßen eine ärztliche Erstmeinung für eine ROP, aber keine nachfolgende Zweitmeinung und ggf. IMST erhalten haben.

## Methoden

### Daten, Studienpopulation und -design

Es wurde retrospektiv eine kontrollierte, nichtrandomisierte Interventionsstudie auf Basis von Versichertenstammdaten, Leistungsabrechnungsdaten der AOK Nordost sowie Daten des Fallmanagementsystems der AOK-Servicecenter durchgeführt. Im Fallmanagementsystem der AOK-Servicecenter werden Versichertenberatungen dokumentiert. Darin wurde erfasst, ob ein Krankenhauseinweisungsschein für eine ROP im AOK-Servicecenter vorgelegt wurde und ob im Zuge dieser Vorlage eine Empfehlung zur RückenSPEZIAL-Teilnahme durch die AOK Nordost erfolgte. Damit war es möglich, eine Grundgesamtheit von Versicherten zu identifizieren, die im Zeitraum vom 05.02.2015 bis zum 30.03.2017 die zwei zentralen Aufgreifkriterien erfüllte:

- Ärztliche Erstmeinung dokumentiert per ROP-Krankenhauseinweisungsschein,
- Beratungsgespräch zur Teilnahme an RückenSPEZIAL (Abb. 1).

Die AOK-Servicecenter-Mitarbeiter prüften vor einer Beratung, ob eine augenscheinlich klar erkennbare Nichteignung für das Zweitmeinungsverfahren vorlag. Versicherte, die binnen 74 Tagen nach der RückenSPEZIAL-Teilnahmeberatung mindestens die „Vorbefundprüfung“ von RückenSPEZIAL in Anspruch genommen hatten, gehörten zur Population *mit* Teilnahme RückenSPEZIAL. Diese Population stellte die Basis für die Interventionsgruppe (IG) dar. Versicherte, die nach der RückenSPEZIAL-Teilnahmeberatung keine RückenSPEZIAL-Leistung in Anspruch genommen hatten, gehörten zur Population *ohne* Teilnahme am Vertrag RückenSPEZIAL. Aus dieser Population wurde im Weiteren die Vergleichsgruppe (VG) gebildet. 74 Tage wurden als Obergrenze für eine beratungsbezogene Teilnahme verwendet, da 95 % aller Tagesdifferenzen zwischen RückenSPEZIAL-Teilnahmeberatung und Erstkontakt (Vorbefundprüfung und/oder IZMV) bei RückenSPEZIAL kleiner gleich 74 Tage waren.

Der Versichertenkontakttag im AOK-Servicecenter war der Stichtag für die Wirksamkeitsbeobachtung in 365 Folgetagen sowie für den Merkmalsabgleich der Vergleichsgruppenziehung in 365 oder 730 Tagen vorab. Patienten ohne durchgängige Versichertenzeit in 730 Tagen vor und 365 Tagen nach Stichtag wurden ausgeschlossen. Für sie waren Vergleichsmerkmale oder Nachbeobachtungszeit nicht vollständig verfügbar. Ebenso mussten 21 RückenSPEZIAL-Teilnehmer ausgeschlossen werden, deren Leistungsdokumentationen aus organisatorischen Gründen nicht vorlagen. Aus diesen bereinigten Populationen von IG und VG wurden in zwei Ziehungsschritten mit exaktem „matching“ und „propensity score matching“ merkmalsgleiche Vergleichspatientenpaare im Verhältnis 1:1 ausgewählt ohne Zurücklegen. Die Analyse befolgte Standards für Sekundärdatenanalyse und Beobachtungsstudien [12, 14].

### Ziehung merkmalsgleicher Vergleichspatienten

Im Ziehungsschritt 1 wurde für zentrale, vor allem rückenschmerzbezogene Merkmale ein exaktes „1:1 matching“ durchgeführt. Je Versicherten aus der bereinigten Population der IG wurde aus der bereinigten Population der VG ein bestgeeigneter Matching-Partner gesucht, der für neun definierte Merkmale eine identische Merkmalskombination aufwies (Tab. 2, Merkmalsabgleich, „1:1“, kursiv hervorgehobene Zeilen): Das Alter wurde mit einer Spannweite von ± 10 Jahre abgeglichen. Einzelkategorien der Merkmale „Bundesland“, „Dauer kontinuierliche quartalsweise ambulante Arztkontakte/Krankenhausaufenthalte mit Rückenschmerzdiagnose“ sowie „Rückenschmerzdiagnose“ wurden teils gemäß Verteilung zusammengefasst, um eine hinreichende Treffermenge zu ermöglichen (Tab. 2). Weitere Variablen wurden dichotom operationalisiert. Mindestens einmaliges Vorkommen des Merkmals regelhaft im Vorjahr bedeutet „1“. Keinerlei Vorkommen des Merkmals regelhaft im Vorjahr bedeutet „0“. Im Ergebnis von Ziehungsschritt 1 gab es IG-Patienten, für die mehrere geeignete VG-Patienten vorlagen. Für diese erfolgte Ziehungsschritt 2.

Für Ziehungsschritt 2 wurden weitere Einflussgrößen als „propensity score“ zusammengefasst. Zur Ermittlung des „propensity score“ wurde mit logistischer Regression für die bereinigten Populationen von IG und VG die Wahrscheinlichkeit geschätzt, zur IG zu gehören, anhand einer erweiterten Liste von Merkmalen für rückenschmerzbezogene Leistungen, psychische Begleiterkrankungen, generelle Krankenhausaufenthalte sowie die Anzahl unterschiedlicher Arzneimittelwirkstoffe (Tab. 2, Merkmalsabgleich, „PS“). Danach wurde für jeden IG-Patienten mit mehreren potenziellen 1:1-VG-Patienten aus Ziehungsschritt 1 derjenige mit dem ähnlichsten „propensity score“ ausgewählt (Nearest-neighbour-Methode).

Für die resultierenden IG-Patienten mit ihren jeweiligen individuell merkmalsgleichen VG-Patienten erfolgte ein Ergebnisvergleich in der Gesamtgruppe sowie in drei für die Evaluation definierten Subgruppen gemäß IZMV-Empfehlung: (i) IZMV und IMST, (ii) IZMV und Regelversorgung, (iii) IZMV und direkte OP-Empfehlung (Abb. 1).

### Rückenschmerzbezogene Kosten und Merkmale

Folgende rückenschmerzbezogene Kosten (RBK) wurden im Beobachtungszeitraum von 365 Tagen ab Stichtag bestimmt: Kosten für stationäre/ambulante Rückenoperationen, Krankenhausaufenthalte mit Hauptentlassdiagnose Rückenschmerz ohne ROP, Schmerzarzneimittel, Physiotherapie, ambulante Facharztbehandlung, Hilfsmittel, ambulante und stationäre Rehabilitation und Krankengeldzahlung. Die Operationalisierung der Kosten und Leistungen wird in Tab. 1 beschrieben. Diese Operationalisierung wurde auch für das „matching“ verwendet für den Zeitraum vor dem Stichtag. Für Versicherte der IG wurden zudem die zusätzlichen Interventionskosten des Vertrags RückenSPEZIAL berechnet.

**Table 1:** 

Leistung/Merkmal	Operationalisierung
*Rückenoperation*	Krankenhausfälle oder Fälle von ambulantem Operieren mit stationärem Aufnahmedatum oder ambulantem Behandlungsdatum im Beobachtungszeitraum und mit OPS-Code-Gruppe 5-83 für Wirbelsäulenoperationen sowie weiteren OPS-Codes für Flavektomie, Laminotomie, Hemilaminektomie und Laminektomie
*Krankenhausfall ohne Rückenoperation*	Krankenhausfälle mit Aufnahmedatum im Beobachtungszeitraum und mit definierten ICD-10-Codes für Rückenschmerz als Hauptentlassdiagnose ohne einen OPS-Code für Rückenoperation
*Schmerzarzneimittel*	Nettokosten für definierte ATC-Codes mit Verordnungsdatum im Beobachtungszeitraum
*Physiotherapie*	Alle Verordnungen mit dokumentiertem Indikationsgebiet Wirbelsäulenerkrankungen 1 und Wirbelsäulenerkrankungen 2 des Heilmittelkatalogs nach § 92 Abs. 6 Satz 1 Nr. 2 SGB V mit Behandlungsdatum im Beobachtungszeitraum
*Ambulante Facharztbehandlung*	Die gesamten Fallkosten für alle Behandlungsfälle mit mindestens einem Behandlungstag des Behandlungsfalls im Beobachtungszeitraum, bei den Arztgruppen Orthopäde, Radiologe, physikalische und rehabilitative Medizin, Neurochirurgie, Neurologie, Neurologie/Psychiatrie, wenn mindestens eine gesicherte Rückenschmerzdiagnose codiert wurde
*Hilfsmittel*	Ausgewählte Gebührenpositionen der Produktgruppen Bandagen, Einlagen, Elektrostimulationsgeräte, Orthesen/Schienen, Schuhe mit Leistungsbeginndatum im Beobachtungszeitraum
*Stationäre Rehabilitation*	Fallkosten der Behandlungsfälle mit Hauptdiagnose oder Operationsdiagnose Rückenschmerz mit Beginndatum im Beobachtungszeitraum
*Ambulante Rehabilitation*	Leistungen zu Rehabilitationssport/Funktionstraining gemäß Reha-Leistungs-Schlüssel mit Leistungsbeginn im Beobachtungszeitraum
*Krankengeld*	Alle Krankengeldfälle mit Beginndatum im Beobachtungszeitraum mit definierten ICD-10-Codes für Rückenschmerz. Krankengeldzahlungen sind einkommensabhängig. Zur Vergleichbarkeit wurden für Interventionsgruppe und Vergleichsgruppe durchschnittliche Krankengeldtageskosten der Interventionsgruppe verwendet (Standardisierung)

Für alle so definierten Leistungen wurde der Zahlbetrag der GKV analysiert ohne etwaige Patientenzuzahlungen (GKV-Perspektive). Der Anstieg der RBK vom Vorjahr auf das Folgejahr in der IG – einschließlich der zusätzlichen Interventionskosten des Vertrags RückenSPEZIAL – wurde mit dem RBK-Anstieg in der VG verglichen. Diese Differenz-von-Differenzen-Methode wurde verwendet, um für Kostenunterschiede im Ausgangsniveau im Vorjahr zu kontrollieren. Aus Gründen der Geheimhaltung wurden Kostenergebnisse lediglich prozentual veranschaulicht.

Das Online-Zusatzmaterial listet eine vollständige Übersicht der verwendeten ICD-10, OPS-Codes etc. für Kosten- und Matching-Variablen.

### Statistische Analyse

Häufigkeitsunterschiede von Gruppenmerkmalen wurden mittels T‑Test für unverbundene Stichproben oder χ^2^-Test beschrieben. Häufigkeitsunterschiede der Ergebnisvariable „Anzahl Patienten mit mindestens einer ROP in 365 Folgetagen“ zwischen IG und VG wurden als relatives Risiko (RR) mit Konfidenzintervallen basierend auf Normalverteilungsannahme veranschaulicht.

Aufgrund der sehr uneinheitlich verteilten teuren ROP-Ereignisse und wegen der pauschalierten Kostenabrechnung für die Leistungen des Vertrags RückenSPEZIAL bei den IG-Patienten war eine Approximation einer Verteilung der RBK nicht möglich. Daher wurde eine Bewertung mittels Bootstrapping-Verfahren und 10.000 Iterationen der Stichprobe durchgeführt. Mit der stichprobenbasierten Verteilung und einem einseitigen Test wurde die gerichtete Hypothese geprüft, dass über den Vertrag RückenSPEZIAL die RBK reduziert werden. Die Analyse erfolgte mit SAS Enterprise Guide V6.1. Das „bootstrapping“ erfolgte mit „proc survey select“ und Ziehen mit Zurücklegen. Als Signifikanzniveau galt α ≤ 0,05.

## Ergebnisse

### Populationsbeschreibung

Aus 135 Patienten der bereinigten Population der Interventionsgruppe konnte für 108 IG-Patienten (80 %) jeweils ein merkmalsgleicher VG-Patient identifiziert werden (Abb. 1). Es wurden 108 IG-Patienten und 108 VG-Patienten verglichen. Aufgrund des exakten Merkmalsabgleichs waren jeweils 55,6 % weiblich. Das Durchschnittsalter betrug 61 Jahre. 53,7 % hatten vor dem Indexquartal für mindestens 8 Quartale kontinuierlich in jedem Quartal eine codierte Rückenschmerzdiagnose. 4,6 % hatten bereits mindestens eine ROP im Vorjahr (Tab. 2).

**Table 2:** 

	Interventionsgruppe (IG, *N* = 108)		Vergleichsgruppe (VG, *N* = 108)		Unterschied IG vs. VG	Merkmalsabgleich	
Merkmale	*n*	%	*n*	%	*p*-Wert^e^	Ziehungsschritt 1	Ziehungsschritt 2
**Soziodemografie**^**a**^							
*Alter Ø (SD) [Median]*	*61,3 (13,1) [61]*		*61,1 (12,6) [61]*		*0,90*	*1:1*	*PS*
*Weiblich*	*60*	*(55,6)*	*60*	*(55,6)*	*1,00*	*1:1*	*PS*
*Pflegestufe ja/nein*	*3*	*(2,8)*	*3*	*(2,8)*	*1,00*	*1:1*	*PS*
**Wohnort**^**a**^							
*Berlin*	*78*	*(72,2)*	*78*	*(72,2)*	*1,00*	*1:1*	*PS*
*Brandenburg*	*30*	*(27,8)*	*30*	*(27,8)*	*1,00*	*1:1*	*PS*
**Versicherungsart**^**a**^							
Familienmitversichert	4	(3,7)	3	(2,8)	n. a.	–	PS
Pflichtversichert	25	(23,1)	28	(25,9)	0,68	–	PS
Rentner	51	(47,2)	51	(47,2)	1,00	–	PS
Arbeitslos	19	(17,6)	18	(16,7)	0,87	–	PS
Freiwillig	6	(5,6)	7	(6,5)	0,78	–	PS
Sonstiges	3	(2,8)	1	(0,9)	n. a.	–	PS
**Dauer kontinuierlicher Rückenschmerzdiagnosen in 2 Vorjahren**^**c**^							
*0 Quartale*	*6*	*(5,6)*	*6*	*(5,6)*	*1,00*	*1:1*	*PS*
*1 Quartal*	*14*	*(13,0)*	*15*	*(13,9)*	*0,85*	*1:1*^*f*^	*PS*
*2 Quartale*	*9*	*(8,3)*	*10*	*(9,3)*	*0,82*		*PS*
*3 Quartale*	*10*	*(9,3)*	*7*	*(6,5)*	*0,47*		*PS*
*4 Quartale*	*4*	*(3,7)*	*5*	*(4,6)*	*n.* *a.*		*PS*
*5 Quartale*	*4*	*(3,7)*	*2*	*(1,9)*	*n.* *a.*		*PS*
*6 Quartale*	*0*	*(0,0)*	*2*	*(1,9)*	*n.* *a.*		*PS*
*7 Quartale*	*3*	*(2,8)*	*3*	*(2,8)*	*n.* *a.*		*PS*
*8 Quartale*	*58*	*(53,7)*	*58*	*(53,7)*	*1,00*	*1:1*	*PS*
**Rückenschmerzdiagnosegruppe 1 im Vorjahr**^**b,c**^							
*Lumbal & zervikal*^*d*^	*61*	*(56,5)*	*61*	*(56,5)*	*1,00*	*1:1*	*PS*
*Zervikal*^*d*^	*4*	*(3,7)*	*2*	*(1,9)*	*n.* *a.*	*1:1*^*f*^	*PS*
*Lumbal*^*d*^	*39*	*(36,1)*	*40*	*(37,0)*	*0,91*		*PS*
*„Missing“*	*4*	*(3,7)*	*5*	*(4,6)*	*n.* *a.*		*PS*
**Rückenschmerzdiagnosegruppe 2 im Vorjahr**^**b,c**^							
Spezifischer Rückenschmerz	97	(89,8)	95	(88,0)	0,89	–	PS
Unspezifischer Rückenschmerz	9	(8,3)	9	(8,3)	1,00	–	PS
„Missing“	2	(1,9)	4	(3,7)	n. a.	–	PS
**Rückenschmerzbezogene Leistungen regelhaft im Vorjahr**^**b**^							
*Rücken-OP Vorvorjahr*	*4*	*(3,7)*	*4*	*(3,7)*	*n.* *a.*	*1:1*	*PS*
*Rücken-OP Vorjahr*	*5*	*(4,6)*	*5*	*(4,6)*	*1,00*	*1:1*	*PS*
Krankenhaus mit rückenbez. Hauptdiagnose ohne ROP	21	(19,4)	15	(13,9)	0,32	–	PS
Bilddiagnostik	43	(39,8)	48	(44,4)	0,60	–	PS
Invasive Verfahren	22	(20,4)	18	(16,7)	0,53	–	PS
Konservative Verfahren	74	(68,5)	72	(66,7)	0,87	–	PS
*Schmerzmittelverordnungen*	*100*	*(92,6)*	*100*	*(92,6)*	*1,00*	*1:1*	*PS*
Physiotherapie	70	(64,8)	63	(58,3)	0,54	–	PS
Facharztkontakt Rückenschmerz	101	(93,5)	104	(96,3)	0,83	–	PS
Hilfsmittel Rückenschmerz	34	(31,5)	31	(28,7)	0,71	Kein Abgleich	
Stationäre Anschlussrehabilitation	3	(2,8)	1	(0,9)	n. a.	–	PS
Ambulante Rehabilitationsleistungen	11	(10,2)	6	(5,6)	0,23	–	PS
**Psychiatrische Diagnosen im Vorjahr**^**b,c**^							
Sucht (F1 ICD-10)	9	(8,3)	11	(10,2)	0,65	–	PS
Schizophrenie (F2 ICD-10)	1	(0,9)	2	(1,9)	n. a.	–	PS
Depression (F3 ICD-10)	46	(42,6)	38	(35,2)	0,38	–	PS
Anhaltende Schmerzstörung (F454 ICD-10)	30	(27,8)	28	(25,9)	0,79	–	PS
Sonstige Diagnosen F ICD-10	54	(50,0)	44	(40,7)	0,31	–	PS
Sonstiger Krankenhausaufenthalt^b^	34	(31,5)	39	(36,1)	0,56	–	PS
Anzahl diverse ATC-3-Steller Vorjahr Ø (SD) [Median]	6,7 (3,5) [7]		6,0 (3,3) [5]		0,26	–	PS
**Arbeitsunfähigkeit**							
*Arbeitsunfähig bei Beobachtungsbeginn*	*18*	*(16,7)*	*18*	*(16,7)*	*1,00*	*1:1*	*PS*
Krankengeldbezug bei Beobachtungsbeginn	11	(10,2)	12	(11,1)	0,83	Kein Abgleich	
Krankengeldanspruch vor Beobachtungsbeginn	34	(31,5)	33	(30,6)	0,90	Kein Abgleich	
**Fakultative Ausschlusskriterien für Programmteilnehmer im Vorjahr**^**b**^							
Wirbelfrakturen	1	(1,9)	5	(4,6)	n. a.	Kein Abgleich	
Parese	0	(0,0)	0	(0,0)	n. a.	Kein Abgleich	
Myasthenie	1	(0,9)	0	(0,0)	0,32	Kein Abgleich	
Krebs	10	(9,3)	19	(17,6)	0,09	Kein Abgleich	
Wirbelsäulentumoren	0	(0,0)	0	(0,0)	n. a.	Kein Abgleich	
Rentenantrag	4	(3,7)	4	(3,7)	n. a.	Kein Abgleich	
**„Propensity score“ **Ø (SD) [Median]	0,19 (0,11) [0,17]		0,17 (0,07) [0,16]		0,26	n. a.	

*Kursiv*: 9 definierte Merkmale des 1:1-Merkmalsabgleichs

*1:1* exakter Merkmalsabgleich, *PS* Merkmalsabgleich mit „propensity score“, Zeilengruppierung entspricht verwendeter Variablengruppierung, *SD* Standardabweichung, *ATC* Anatomisch-Therapeutische Wirkstoffklasse, *n.* *a.* nicht anwendbar, z. B. da Fallzahl < 5, *KH* Krankenhaus

^a^Letzte Versichertenangabe im Kalenderjahr des Beobachtungsbeginns

^b^Im genannten Zeitraum wurde ≥ 1 betreffende Diagnose/Leistung codiert/abgerechnet

^c^Mindestens eine ambulant gesicherte Diagnose oder stationäre Hauptentlassdiagnose ICD-10

^d^Diagnosegruppierung nach Lokalisationsangaben an fünfter Stelle ICD-10-Code sowie ohnehin spezifische ICD-10-Code-3-Steller

^e^Bei Häufigkeitsangaben χ^2^-Test mit einem Freiheitsgrad, bei Durchschnittswerten T‑Test für 2 unverbundene Stichproben

^f^Kategorie der Variable wurde gruppiert über mehrere Ausprägungen

Neben diesen ausgewählten exakt abgeglichenen Merkmalen hatten im Vorjahr 93,5 % der IG-Patienten mindestens einmal eine rückenschmerzbezogene ambulante Facharztbehandlung (VG 96,3 %), 64,8 % mindestens eine Physiotherapieleistung (VG 58,3 %), 20,4 % mindestens eine invasive Schmerztherapie/periradikuläre Therapie (VG 16,7 %), 19,4 % mindestens einen Krankenhausaufenthalt mit Hauptentlassdiagnose Rückenschmerz ohne ROP (VG 13,9 %, Tab. 2).

Bei IG-Patienten erfolgten die Vorbefundprüfung und die IZMV im Median 6 Tage nach dem Servicecenterkontakt (Durchschnitt 6,18 Tage, SD 13,90 Tage). In dieser Zwischenzeit fand regelhaft keine ROP statt.

### Weniger Patienten mit Rückenoperation

**Figure 2:**
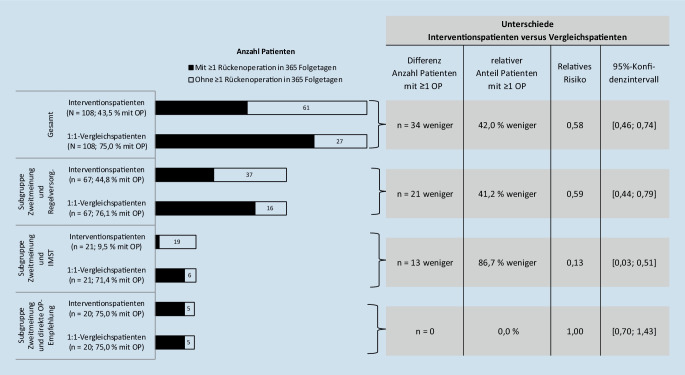

In 365 Folgetagen hatten 47 IG-Patienten (43,5 %) und 81 VG-Patienten (75,0 %), also 42,0 % weniger IG-Patienten, mindestens eine ROP (relatives Risiko [RR] 0,58; *p* < 0,001). Eine Betrachtung in drei Subgruppen zeigte:

- 20 IG-Patienten (18,5 %) erhielten beim IZMV eine direkte OP-Empfehlung.
- 21 IG-Patienten (19,4 %) erhielten zusätzlich zum IZMV auf ärztliche Empfehlung eine IMST. Von diesen IG-Patienten hatten 86,7 % weniger mindestens eine ROP, im Vergleich zu ihren individuell merkmalsgleichen VG-Patienten (RR 0,13; *p* < 0,001, *n* = 21).
- 67 IG-Patienten (62,0 %) erhielten „alleinig“ IZMV. Von diesen IG-Patienten hatten 41,2 % weniger mindestens eine ROP im Vergleich zu ihren individuell merkmalsgleichen VG-Patienten (RR 0,59; *p* < 0,001; *n* = 67, Abb. 2).

### Weniger rückenschmerzbezogene Kosten

**Figure 3:**
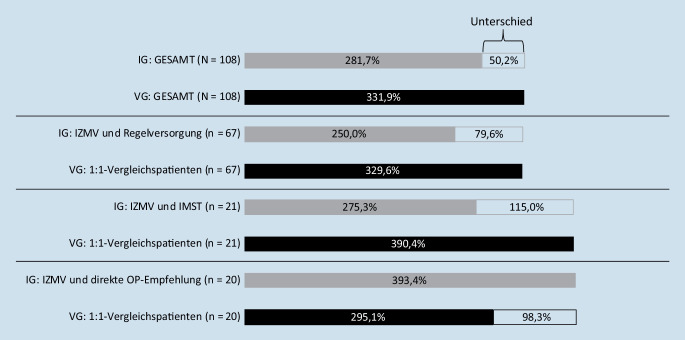

Bei IG-Patienten stiegen die RBK – einschließlich der zusätzlichen Interventionskosten für den Vertrag RückenSPEZIAL – um durchschnittlich 50,2 %-Punkte weniger stark an als bei den VG-Patienten (*p* = 0,088). Dieser positive Effekt war noch stärker ausgeprägt in der Subgruppe der IG-Patienten, die nach IZMV auf ärztliche Empfehlung eine kostenintensive IMST erhielten (*n* = 21; durchschnittlich 115,0 %-Punkte niedrigerer Anstieg der RBK; *p* = 0,049). Patienten mit alleinigem IZMV hatten einen durchschnittlich 79,6 %-Punkte niedrigeren Anstieg der RBK gegenüber ihren individuell merkmalsgleichen VG-Patienten (*n* = 67; *p* = 0,042, Abb. 3). Die Unterschiede der RBK waren in den genannten Subgruppen signifikant, für die Gesamtgruppe allerdings knapp nicht.

**Figure 4:**
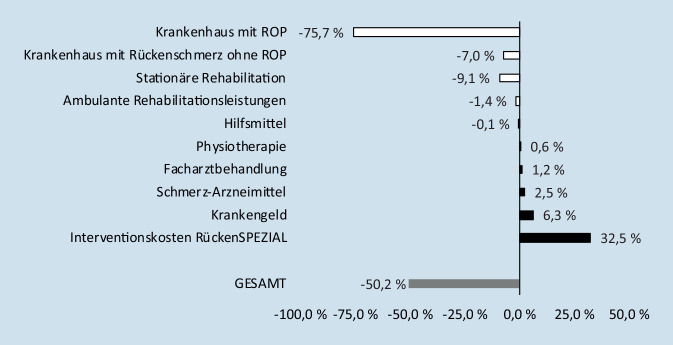

Der insgesamt niedrigere Anstieg der RBK ging im Wesentlichen zurück auf anteilig weniger IG-Patienten mit Krankenhauskosten für ROP im Folgejahr. IG-Patienten hatten allerdings in den Leistungsbereichen Krankengeld, Schmerzmittel, Facharztbehandlung und Physiotherapie durchschnittlich einen geringfügig höheren Anstieg der RBK als die VG-Patienten (Abb. 4).

## Diskussion

Basierend auf einer Grundgesamtheit von Versicherten mit ROP-Erstmeinung und Krankenhauseinweisungsschein, identifiziert in den AOK-Servicecentern, erfolgte eine Matched-pairs-Analyse mit GKV-Daten. Vergleichsversicherte wurden aus dieser Grundgesamtheit gewonnen, die trotz Angebot durch die Krankenkasse nicht an dem Vertrag RückenSPEZIAL teilgenommen hatten. Die Analyse zeigte bei RückenSPEZIAL-Teilnehmern 42,0 % weniger Patienten mit mindestens einer Rückenoperation und 50,2 %-Punkte weniger Anstieg rückenschmerzbezogener Kosten im Folgejahr.

### Weniger Patienten mit Rückenoperation

Einarmige Fallbeobachtungen aus den USA berichteten aus ärztlichen Zweitmeinungseinschätzungen eine theoretische ROP-Vermeidbarkeit von 17,2 % [4], 44,5 % [6] oder 60,7 % [3]. In Deutschland wurde eine ROP-Ablehnquote von 92,3 % bei Zweitmeinungen zu bereits terminierten Operationen berichtet [13].

Bei 5 von 20 IG-Patienten mit ROP-Bestätigung im IZMV erfolgte in 365 Folgetagen *keine* ROP (Abb. 2, Subgruppe Zweitmeinung und direkte OP-Empfehlung). Selbst bei ROP-Bestätigung im aufwendigen Zweitmeinungsverfahren entschied sich ein relevanter Anteil von Patienten im Nachgang dennoch gegen eine ROP. Eine ROP-Bestätigung oder -Ablehnung am Zweitmeinungstag ist scheinbar nur ein kurzfristig gültiger Endpunkt.

Ein belastbarerer Endpunkt ist eine datenbasiert vollständige Analyse von ROP für 365 Folgetage. Das wurde in Deutschland bereits mit GKV-Daten untersucht: Nach 92,3 % OP-Ablehnungen am Zweitmeinungstag erfolgte bei 79,0 % der Zweitmeinungsteilnehmer in 12 Folgemonaten dauerhaft keine ROP [13]. Die vorliegende Studie ermittelte analog: Nach 81,5 % OP-Ablehnungen erfolgte bei 56,5 % der Zweitmeinungsteilnehmer in 12 Folgemonaten dauerhaft keine ROP (Abb. 2, Gesamt 100,0–43,5 %). Der Unterschied bei ROP-Ablehnungen und dauerhafter ROP-Vermeidung in dieser Studie kann vermutlich mit einem 6 Jahre höheren Populationsdurchschnittsalter, ggf. entsprechend längerer Schmerzchronifizierung, abweichender Versichertenstruktur einer anderen Krankenkasse und ggf. auch mit abweichender Interventionsintensität erklärt werden.

Diese Studie verwendete erstmalig in Deutschland eine Matched-pairs-Vergleichsgruppe und zeigte weiterhin: Auch 25,0 % der Vergleichspatienten hatten in 12 Folgemonaten nach Ausstellung eines Krankenhauseinweisungsscheins *keine* ROP. Es ist möglicherweise denkbar, dass in einigen dieser Fälle *vor* der Ausstellung eines Krankenhauseinweisungsscheins für eine teure risikobehaftete ROP weitere alternative Maßnahmen erwägbar gewesen wären.

Mit der Intervention RückenSPEZIAL war eine Wirksamkeit von 42,0 % *weniger ROP als in der Vergleichsgruppe* assoziiert. Eine Betrachtung *alleinig* der Zweitmeinungsteilnehmer mit 56,5 % (Abb. 2, Gesamt 100,0–43,5 %) oder 79,0 % [13] dauerhaft ausgebliebenen ROP kann zur Überschätzung der Wirksamkeit eines Zweitmeinungsverfahrens verleiten, *wenn* die Vergleichspatienten nicht beachtet werden, die auch ohne Zweitmeinungsverfahren dauerhaft keine ROP hatten.

Es zeigte sich ein Hinweis, dass eine vergleichsweise ROP-Vermeidung bei IG-Patienten mit „IZMV und IMST“ höher ausfällt als bei Patienten mit „IZMV und Regelversorgung“. Für IMST wurden bereits positive Effekte auf die Wiederherstellung der Arbeitsfähigkeit ermittelt [15]. Die vorliegende Evaluation zeigte nun für IMST eine mögliche positive Assoziation mit ROP-Vermeidung. Diese Assoziation ist jedoch zumindest anteilig auch darauf zurückzuführen, dass Ärzte per IZMV-Empfehlung IMST-geeignete Patienten auswählten. Offenbar gibt es spezifisch geeignete Versicherte, bei denen IMST eine ROP abwenden kann. Eine nähere Beschreibung spezifischer Eignungskriterien könnte eine frühzeitige Identifikation dieser Patienten unterstützen.

### Kosteneffizienz dank hoher ROP-Wahrscheinlichkeit

Für die Intervention des Vertrags RückenSPEZIAL gibt es aus GKV-Perspektive einen Hinweis auf Kosteneffektivität hinsichtlich der RBK, auch wenn das definierte Signifikanzniveau nicht erreicht wurde. Aufgrund der stringent gewählten Aufgreifkriterien gab es in der Studienpopulation eine hohe Wahrscheinlichkeit für ein tatsächlich zukünftig eintretendes, teures ROP-Ereignis. Damit konnte die Refinanzierung der zusätzlichen Interventionskosten des Vertrags RückenSPEZIAL maßgeblich durch ROP-Vermeidung gelingen. Studien zu Kosteneffekten für ein Zweitmeinungsverfahren bei ROP und für RBK konnten nicht gefunden werden [11]. Laut einer Studie aus dem Jahr 1982 auf Basis US-amerikanischer Abrechnungsdaten waren jedoch Zweitmeinungen bei einigen anderen elektiven Operationen kosteneffektiv [10].

Seit dem 19.11.2021 hat jeder gesetzlich Versicherte nach ärztlicher Indikationsstellung eines Eingriffs an der Wirbelsäule Anspruch auf ein Zweitmeinungsverfahren [5]. Beim besonderen Versorgungsprogramm RückenSPEZIAL wurden für die Wirksamkeit bei dauerhafter ROP-Vermeidung oder Kosteneffizienz zwei Erfolgsfaktoren deutlich, die für die konkrete Umsetzung oder Weiterentwicklung des generellen gesetzlichen Anspruchs auf ein Zweitmeinungsverfahren informativ und relevant sein können:

(1) Das Aufgreifkriterium für Zweitmeinung fokussierte auf einen eingegrenzten Kreis von Patienten, die bereits begonnen hatten, ihre ROP konkret organisatorisch vorzubereiten (Vorlage des Krankenhauseinweisungsscheins im AOK-Servicecenter). Das gewährleistete eine hohe ROP-Eintritts-Wahrscheinlichkeit und damit eine hohe Chance auf ROP-Vermeidung. (2) Das Zweitmeinungsverfahren erfolgte stets fachübergreifend und leitete direkt in fachübergreifend ermittelte konservative Behandlungsalternativen über bzw. leitete bei festgestellter Eignung direkt die umfangreiche konservative Maßnahme IMST ein.

### Stärken

Zu den Stärken dieser GKV-Daten-Analyse zählt, dass sie teils erstmalig in Deutschland

- systematisch auf Daten zu ROP-Erstmeinungen zurückgreifen konnte,
- Rückenoperationen vollständig datenbasiert für 365 Tage nachbeobachtete,
- die Programmwirksamkeit mittels Matched-pairs-Vergleichsgruppe untersuchte und hinsichtlich ROP-„Vermeidung“ transparent machte, dass auch Vergleichspatienten teils keine ROP hatten,
- spezifisch rückenschmerzbezogene Merkmale und Kosten analysierte,
- eine stichtagsbezogene tagesgenaue Datenanalyse realisierte.

Die Analyse krankheitsspezifischer Kosten ermöglichte eine Verringerung von Verzerrungen durch möglicherweise nicht krankheitsassoziierte Kosten bspw. für eine Herzerkrankung.

### Limitationen

Vergleichsversicherte wurden aus einer Grundgesamtheit von Patienten mit ROP-Krankenhauseinweisungsschein gewonnen, die trotz des beratenden Angebots durch die Kasse nicht an dem Vertrag RückenSPEZIAL teilgenommen hatten. Entsprechend ist von einem Selbstselektionseffekt auch bei der Vergleichsgruppe auszugehen. In der Versorgungsrealität entscheidet sich der Patient für oder gegen eine Teilnahme am Vertrag RückenSPEZIAL und nicht eine Randomisierung. Diese Selbstselektion – RückenSPEZIAL-Teilnehmer hatten vermutlich eine erheblich kritischere Einstellung zur ROP – ist damit ein nicht näher differenzierbarer Bestandteil des unter realen Versorgungsbedingungen ermittelten Effekts.

Der Selbstselektionseffekt kann mit den vorliegenden Daten nicht weiter adjustiert werden. Die Daten ermöglichten jedoch grundlegend ein detailliertes „matching“ rückenschmerzbezogener Merkmale. Allerdings konnten nicht alle datenverfügbaren Merkmale mit der Matched-pairs-Methode bestmöglich adjustiert werden. Für drei Merkmale verblieben erkennbare Häufigkeitsunterschiede zwischen IG und VG (Tab. 2):

1. IG-Patienten hatten häufiger einen rückenschmerzbezogenen Krankenhausaufenthalt ohne ROP im Vorjahr.
2. IG-Patienten hatten häufiger Depressionsdiagnosen.
3. IG-Patienten hatten seltener nichtwirbelsäulenassoziierte Krebsdiagnosen.

In der Gesamtschau dieser und weiterer geringfügiger Häufigkeitsunterschiede wird angenommen, dass daraus resultierende Verzerrungen oder mögliche Über‑/Unterschätzungen des Effekts sich tendenziell gegenseitig ausbalancieren. Weitere Ergebnisverzerrungen können nicht ausgeschlossen werden, falls relevante, aber nicht (vollständig) verfügbare Merkmale wie etwa die Schmerzklassifikation nach von Korff, Sportaktivität oder Beruf ungleich verteilt sind. Weiterhin können bislang unbekannte Störgrößen die Effektschätzer verzerren.

## Fazit für die Praxis

- Mit besonderen Dokumentationsdaten zu AOK-Beratungen konnten Erstempfehlungen zu Rückenoperationen einheitlich erfasst werden. Dies ermöglichte – mit den genannten Limitationen – die erste kontrollierte Interventionsstudie in Deutschland für ein Versorgungsprogramm mit Zweitmeinungsverfahren bei empfohlenen Rückenoperationen.
- Bei Patienten, die sich im realen Versorgungsalltag für eine Teilnahme am Zweitmeinungsverfahren entschieden, hatten im Folgejahr 42 % weniger eine Rückenoperation als bei Vergleichspatienten, die sich gegen eine Teilnahme entschieden.
- Patienten, die nach der Zweitmeinung auf ärztliche Empfehlung noch intensive Betreuung mit interdisziplinär-multimodaler Schmerztherapie erhielten, hatten den geringsten Anteil an Rückenoperationen im Folgejahr.
- Aus Perspektive der gesetzlichen Krankenversicherung erscheint das Versorgungsprogramm RückenSPEZIAL kosteneffizient, maßgeblich aufgrund von Kosteneinsparungen bei Rückenoperationen.
- Das Programm ist praxistauglich sowohl mit als auch ohne zusätzliche interdisziplinär-multimodale Schmerztherapie für eine spezifische Population mit bereits ausgestelltem Krankenhauseinweisungsschein für eine Rückenoperation.

## Supplementary Information





## Abkürzungen

GKV: Gesetzliche Krankenversicherung
IG: Interventionsgruppe
IMST: Interdisziplinär-multimodale Schmerztherapie
IZMV: Interdisziplinäres Zweitmeinungsverfahren
RBK: Rückenschmerzbezogene Kosten
ROP/OP: Rückenoperation
VG: Vergleichsgruppe

## References

[1] Ali J, Pieper D (2017). Kaum aktuelle Daten zu Zweitmeinungsverfahren vorhanden – eine systematische Ubersichtsarbeit. Gesundheitswesen.

[2] Destatis (2019) Fallpauschalenbezogene Krankenhausstatistik (DRG-Statistik) Operationen und Prozeduren der vollstationären Patientinnen und Patienten in Krankenhäusern (4 Steller) 2018. https://www.destatis.de. Zugegriffen: 12. Okt. 2021

[3] Epstein NE (2013). Are recommended spine operations either unnecessary or too complex? Evidence from second opinions. Surg Neurol Int.

[4] Epstein NE, Hood DC (2011). “Unnecessary” spinal surgery: a prospective 1-year study of one surgeon’s experience. Surg Neurol Int.

[5] G-BA (2021). Beschluss des Gemeinsamen Bundesausschusses über eine Änderung der Richtlinie zum Zweitmeinungsverfahren (Zm-RL): Aufnahme von Eingriffen an der Wirbelsäule in den Besonderen Teil der Richtlinie.

[6] Gamache FW (2012). The value of “another” opinion for spinal surgery: a prospective 14-month study of one surgeon’s experience. Surg Neurol Int.

[7] Marnitz U (2020). Die interdisziplinäre, multimodale Schmerztherapie in der konservativen Wirbelsäulentherapie. Wirbelsäule.

[8] Nürnberg V, Meier M-T (2021). Patientenrecht Zweitmeinung.

[9] Pieper D, Hess S, Mathes T (2018). Bestandsaufnahme zu Zweitmeinungsverfahren in der Gesetzlichen Krankenversicherung (GKV). Gesundheitswesen.

[10] Ruchlin HS, Finkel ML, McCarthy EG (1982). The efficacy of second-opinion consultation programs: a cost-benefit perspective. Med Care.

[11] S-V-R (2018). Sachverständigenrat zur Begutachtung der Entwicklung im Gesundheitswesen: Gutachten 2018, Bedarfsgerechete Steuerung der Gesundheitsversorgung.

[12] Swart E, Bitzer EM, Gothe H (2016). A consensus German reporting standard for secondary data analyses, version 2 (STROSA-STandardisierte BerichtsROutine fuer SekundaerdatenAnalysen). Gesundheitswesen.

[13] Überall MA, Müller-Schwefe GH, Nolte T (2020). Chronische Rückenschmerzen: operieren oder nicht?. Schmerzmedizin.

[14] von Elm E, Altman DG, Egger M (2014). The strengthening the reporting of observational studies in epidemiology (STROBE) statement: guidelines for reporting observational studies. Int J Surg.

[15] Wagner CJ, Ayyad G, Otzdorff A (2019). Behandlungs- und Kosteneffekte der interdisziplinaren multimodalen Schmerztherapie bei Patienten mit Ruckenschmerz: Eine kontrollierte, nicht-randomisierte Interventionsstudie mit GKV-Daten und Teilnehmerbefragung. Schmerz.

